# Impact of Shaking EDTA, Citrate, or MgSO_4_ Tubes on Platelet Count Results

**DOI:** 10.3390/jcm13185350

**Published:** 2024-09-10

**Authors:** Michel Soulard, Hela Ketatni, Claire Visseaux, Pascale Croix, Patrick Cohen

**Affiliations:** 1 Biogroup Hematology Laboratory, La Chocolaterie, 92300 Levallois Perret, France; hela.ketatni@biogroup.fr (H.K.); claire.visseaux@biogroup.fr (C.V.); pascale.croix@biogroup.fr (P.C.); 2 Division of Laboratory Medecine, Diagnostic Department, Geneva University Hospitals and Faculty of Medicine, 1205 Geneva, Switzerland; patrickraoul.cohen@hug.ch

**Keywords:** platelets, pseudothrombocytopenia, anticoagulants

## Abstract

**Background**: In EDTA-induced pseudothrombopenia, citrate or MgSO_4_ are recommended for platelet counting. Pre-analytical conditions are poorly defined for tubes containing MgSO_4_ or citrate. In this study, we analyzed the impact of agitation of these tubes on platelet counts. **Methods:** K2EDTA, citrate, and MgSO_4_ tubes from 70 patients were gently agitated on a wheel rotating at 20 rpm. Platelets were analyzed on the Sysmex XN analyzer at different times, and the percentage of platelet deviation from T0 was assessed and compared with the desirable bias of the EFLM. **Results**: at 180 min in fluorescence, the relative variation of platelets after shaking is 1.17% for K2EDTA, −29.76% for citrate, and −33.18% for MgSO_4_, while for unshaken MgSO_4_ platelets the variation is −1.3%. The reduction in platelet numbers when citrate or MgSO_4_ tubes are shaken is linked to the appearance of platelet clusters. **Conclusions**: agitation of MgSO_4_ and especially citrate tubes led to a decrease in platelet counts due to the formation of platelet aggregates; on the other hand, platelet counts on EDTA are virtually stable. During transport, we recommend putting sodium citrate and MgSO_4_ tubes in an upright position and avoiding shaking them to avoid giving an erroneous platelet result.

## 1. Introduction

EDTA-dependent pseudothrombocytopenia (PTP) is characterized by the presence of EDTA-induced platelet clusters, which disappear when anticoagulants other than EDTA are used [1]. PTP represents a challenge because of the risk of providing an erroneous platelet count result and hence an inaccurate diagnosis, with potentially serious consequences for the patient. The prevalence of PTP ranges from 0.01 to 1%, depending on the study [1,2,3]. PTP is a temperature- and time-dependent phenomenon. It is linked to the existence of mainly IgG autoantibodies (IgG1) directed against cryptic epitopes of GpIIbIIIa unmasked by calcium chelation with EDTA [1,2,4].

Sodium citrate is most often used, but platelet counts must be taken within 3 h of sampling. Furthermore, since this anticoagulant is in liquid form, the platelet result must be corrected, and the conversion factor is 1.1 [1].

Magnesium sulfate (MgSO_4_) is also recommended for platelet counts in PTP.

The anticoagulant effect of MgSO_4_ results from the inhibition of fibrinogen binding to the GpIIbIIIa complex and inhibition of thromboxane A2 formation, on the one hand, and from the cAMP formation, which blocks platelet activation by inhibiting intra-tubular calcium mobilization, on the other [4,5,6]. MgSO_4_ is stable for 12 h [2] and, with the Sysmex XN in fluorescence, shows a bias of −2.03% compared with K2EDTA, which is 5 times lower than that of sodium citrate compared with K2EDTA [7].

Pre-analytical recommendations for transporting citrate or MgSO_4_ tubes for platelet counting are not known. The aim of this study was to determine the impact of standardized agitation of K2EDTA, sodium citrate, and MgSO_4_ tubes on platelet count results. To this end, K2EDTA, sodium citrate, and MgSO_4_ tubes from 70 patients with no initial platelet clusters were slowly rotated for platelet analysis at different times on the Sysmex XN analyzer in impedance and fluorescence. Stability was assessed by comparing the percentage deviation of platelets from the initial time to the EFLM (European Federation of Clinical Chemistry and Laboratory Medicine) bias [8].

## 2. Material and Methods

We collected blood samples from 87 patients (45 males; 42 females; median: 63 years; min: 19 years; max: 93 years) in our laboratory. These samples were collected with 3 tubes and in the following order: first a K2EDTA (EDTA) tube, 7.2 mg, 4 mL, BD Vacutainer; then sodium citrate 0.109 M, 2.7 mL, BD Vacutainer (Becton Dickinson; Franklin Lakes, NJ, USA); finally MgSO_4_, 33.8 μmol, 2.7 mL, S-Monovette Thromboexact (Sarstedt; Numbrecht, Germany).

The experiment is carried out at a temperature between 15 °C and 25 °C. Within a maximum of 30 min of sampling corresponding to T0, platelets from all 3 tubes are analyzed, in duplicate in open mode, on the Sysmex XN10 (version 22.16) in impedance and fluorescence (oxazine).

For each patient, 500 µL of each citrate and MgSO_4_ tube was kept in the vertical position as a control. Blood smears were stained with SP10, then read using a Zeiss light microscope (Primo Star) with an X50 objective (Zeiss, Oberkochen, Germany). The presence of platelet clusters corresponds to the observation of at least 5 agglutinated platelets per field in more than 3 out of 30 fields. Among the 30 fields examined, 10 were on the lateral edges of the smear, 10 in the monolayer, and 10 in the tail. Of the 87 patients, 17 were excluded because of platelet clusters. For each of the 70 patients with no platelet clusters on any of the 3 anticoagulants, the 3 tubes were placed on a flat disc 20 cm in diameter, inclined at 45° and rotating continuously at a speed of 1 revolution every 3 s: 20 revolutions (revs) per minute (rpm). After 15 min, 30 min, 60 min, 90 min, 120 min, 150 min, and 180 min, for each patient, the 3 tubes were simultaneously removed from the wheel and placed in a vertical position for 10 min, during which time the tubes were analyzed one by one in duplicate after being shaken by 10 successive inversions. At the end of the analysis, the 3 tubes were placed simultaneously on the wheel to be shaken. Considering that for a replica the maximum difference between the 2 values was 10%, no points were rejected. Platelet between-run imprecision during the study period is shown in Table S1. Platelet results on citrate were multiplied by 1.1.

The averages derived from each duplicate were calculated at different shaking times, and the percentage difference ratios (PD%) were evaluated according to PD% = (Tx − T0)/ T0 × 100 with the Tx mean platelet result for the sample considered at time x; the T0 average at T0 for the same sample. The mean and pseudo median (Hodges-Lehmann estimator) (PM) and their 95% CI were calculated.

The impact of agitation on platelet stability was assessed by comparing PM of PD (%) (PMed) to the desirable platelet bias derived from EFLM (European Federation of Clinical Chemistry and Laboratory Medicine Biological Variation) [8] with equivalence tests.

*p*-values are calculated either with a two-side test (Wilcoxon) to test equivalence with respect to the desirable EFLM bias (4.5%) if PMed is less than the bias or with a 2-side sign test (Wilcoxon) if PMed is greater than the bias.

In addition, regression lines for the mean of PD (%) as a function of the number of revolutions by least-squares fitting were established with intercept (OLS) and without intercept (RTO). R^2^ for RTO was calculated according to Eisenhauer [9].

The Shapiro–Wilk test was used to analyze the normality of the distribution of results.

The confidence interval for the mean is calculated using Student’s *t*-test, and for the pseudomedian using Tukey’s test. The significance level is *p* < 0.05. All statistical analyses were performed with MedCalc^®^ Statistical Software version 23.0.2 (Ostend, Belgium).

## 3. Results

### 3.1. EDTA

At T0, the mean platelet fluorescence of the 70 patients on K2EDTA was 273.3 × 10^9^/L 95% CI (242.2; 304.4), the pseudomedian 261 × 10^9^/L 95%CI (242.5; 278.5), the minimum 19 × 10^9^/L, and the maximum 990 × 10^9^/L (Table S2).

After shaking with EDTA, the PMed of platelets in impedance increased progressively from 0.65% at 300 revs to 2.26% at 3600 revs, while it rose for platelets in fluorescence from 0.38% to 1.17%. In impedance and fluorescence, the upper limit of the 95 CI of the PMed at 3600 revs was below the EFLM desirable bias of 4.5% of platelets (Table 1).

**Figure 1:**
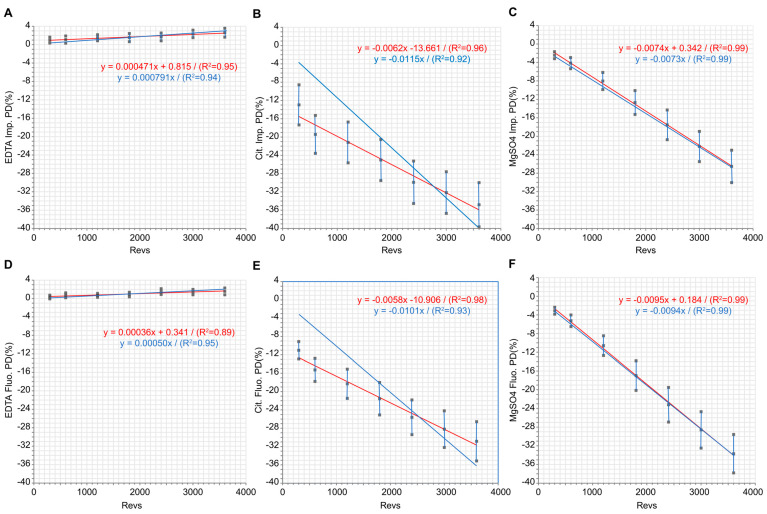
Plot of the percentage difference in platelet averages (K2EDTA, citrate, or MgSO_4_) of 70 patients measured on XN10 (in impedance or fluorescence) compared with T0: PD (%) as a function of the number of revolutions obtained by shaking on a 20 cm diameter disc at 20 rpm. (**A**) K2EDTA impedance; (**B**) citrate impedance; (**C**) MgSO_4_ impedance. (**D**) K2EDTA fluorescence; (**E**) citrate fluorescence; (**F**) MgSO_4_ fluorescence. In red are the least squares regressions not passing through the origin (OLS); in blue are the regressions passing through the origin (RTO). Vertical lines join the 95%CI limits of each PD mean. **Abbreviations:** R^2^: determination coefficient.

Regression analysis of the mean PD% of EDTA platelets as a function of the number of turns showed straight lines with very small slopes differing little in impedance: 47 × 10^−5^ (34–60) for OLS, 79 × 10^−5^ (58–99) for RTO, and in fluorescence 36 × 10^−5^ (21–52) for OLS and 50 × 10^−5^ (39–61) for RTO (Figure 1A,D).

### 3.2. Citrate

For citrate, impedance and fluorescence showed a significant and continuous decrease in platelet numbers during agitation (Table 1). In impedance, PMed went from −12.36% for 300 revs to −33.92% for 3600 revs, and in fluorescence, from −10.55% for 300 revs to −29.76% for 3600 revs. For citrate 300 turns, PD dispersion was significant but more marked in impedance 95%CI PMed (−16.96%; −7.37%) than in fluorescence 95%CI PMed (−12.44%; −8.29%) (Table 1). PMed max for 300 turns in fluorescence was −35.4%.

By comparison, the PMed of platelets from citrate control tubes held for 3 h in a vertical position without agitation was −15.6%.

From 300 revs, impedance and fluorescence PMed exceeded the EFLM desirable (4.5%) and minimal (6.7%) biases of platelets.

Comparison of regression plots of the mean PD% of impedance citrate or fluorescence citrate platelets against revs showed OLS lines whose slopes differed little −62 × 10^−4^ 95% CI (−76; −48) in impedance and −58 × 10^−4^ 95% CI (−66; −49) in fluorescence (Figure 1B,E). In contrast, the RTOs showed slopes two times higher than the previous ones: −115 × 10^−4^ 95% CI (−149; −82) in impedance and −101 × 10^−4^ 95% CI (−127; −74) in fluorescence (Figure 1B,E). A correlation between the percentage of citrate slides showing platelet clusters at different agitation times and the mean number of platelets measured at these times showed R = −0.93 (−0.99, −0.67) for impedance and R = −0.87 (−0.97, −0.42) for fluorescence.

Consequently, during agitation, a rapid decrease in the number of platelets on citrate was observed, linked to the appearance of platelet clusters. This decrease is −101 × 10^−4^% per revolution in fluorescence and −115 × 10^−4^% in impedance.

### 3.3. MgSO_4_

For MgSO_4_, impedance and fluorescence showed a progressive decrease in platelet numbers during agitation, which was less rapid and less dispersed than for citrate. PMed impedance rose from −2.42% at 300 revs to −25.49% at 3600 revs, and PMed fluorescence, from −2.66% at 300 revs to −33.18% at 3600 revs (Table 1).

By way of comparison, the PMed of platelets from control MgSO_4_ tubes kept in a vertical position without agitation for 3 h of our study was −0.85%.

In contrast to citrate, the slopes of the regression plots of the mean PDs of MgSO_4_ platelets in fluorescence differed little between the RTO regression −94 × 10^−4^ 95% CI (−96; −92) and the OLS regression −95 × 10^−4^ 95% CI (−99; −91)). Slopes were slightly lower in impedance: −73 × 10^−4^ 95%CI (−75; −71) for RTO and −74 × 10^−4^ 95% CI (−78; −71) for OLS (Figure 1C,F). Correlation between the percentage of slides on MgSO_4_ showing platelet clusters at different agitation times versus the number of platelets measured at these different times showed in impedance R = −0.83 (−0.98; −0.57) and in fluorescence R = −0.89 (−0.99; −0.73).

During agitation, a decrease in platelet numbers on MgSO_4_ was observed, linked to the appearance of platelet clusters. This decrease was −94 × 10^−4^% per revolution in fluorescence and −73 × 10^−4^% in impedance.

## 4. Discussion

This study showed that shaking tubes at 20 rpm at room temperature resulted in an early decrease in platelet counts on citrate and a later decrease on MgSO_4_. Platelet counts on EDTA, on the other hand, showed little change.

For K2EDTA, after 3 h of agitation, the platelet PMed in fluorescence was 1.17% 95% CI (0.88–1.68) and in impedance 2.26% 95% CI (1.50–3.07). Although statistically significant (Wilcoxon test: *p* = 0.009), these differences are still both below the desirable bias (4.5%) and the desirable imprecision (3.6%) of EFLM. Plots of mean platelet PD as a function of number of turns show slopes that, although slightly different (79 10^−5^ 95% CI (62–96)10^−5^ for impedance and 50 10^−5^ 95 % CI (39–61)10^−5^ for fluorescence) remain very low. All in all, for K2EDTA tubes, shaking for 3 h at 20 rpm at room temperature had very little impact on platelet results, with variations close to the coefficient of variation obtained in the laboratory for the normal platelet control level (2.1%).

Before shaking, platelet results correlated well between K2EDTA and MgSO_4_ in fluorescence mode only. Indeed, the mean relative bias of MgSO_4_ compared with K2EDTA was −2.9% 95% CI (−2.2; −3.7) in fluorescence and −10.5% 95% CI (−9.6; −11.3) in impedance. These results corroborated those previously published with the Sysmex XN analyzer [7].

After shaking the K2EDTA and MgSO_4_ tubes for 3 h, the mean relative bias of MgSO_4_ compared with K2EDTA was −36.5% 95%CI (−34.5; −38.5) in fluorescence and −36.9% 95%CI (−35.3; −38.5) in impedance. These results show that the platelet results on MgSO_4_, which were initially well correlated with K2EDTA in fluorescence, are no longer correlated after 3 h of agitation, whatever the analysis mode.

For tubes containing citrate or MgSO_4_, agitation is responsible for platelet depletion since, at 3 h in fluorescence, the PMed of citrate tubes held upright was −15.6%, while the PMed of the same tubes subjected to agitation was −29.7%. For MgSO_4_, the PMed varied from −0.85% for unstirred tubes to −33.18% for stirred tubes. It is worth noting that citrate-containing tubes held in an upright position without agitation show a −15.6% decrease after 3 h, far exceeding the 4.5% desirable bias and even the 10.5% desirable total error of the EFLM. Consequently, in our study conducted at room temperature, the stability of platelets collected in citrate tubes kept in a vertical position without agitation is less than 3 h. On the other hand, under the same conditions, platelets sampled on MgSO_4_ remain stable for 3 h.

Following agitation of citrate or MgSO_4_ tubes, platelets are reduced by the appearance of platelet clusters. In fact, the decrease in platelets observed during agitation of citrate or MgSO_4_ tubes correlates with the increase over time in the percentage of smears made from these anticoagulants showing platelet clusters according to the criterion defined: for a slide, clusters are present if there are more than 5 clumped platelets per field in more than 3 fields out of 30.

With citrate, the sharp drop in platelet numbers as early as 15 min (300 revs), reflected by the difference in RTO versus OLS slopes—RTO slopes are about twice as high as OLS slopes—could be explained by the existence of small aggregates of two to four platelets not initially counted under the microscope because of the definition of platelet clusters comprising at least five platelets [2]. The observation in certain patients of the presence of small platelet clusters on citrate within 30 min after collection at room temperature, independently of any agitation, has already been reported in the literature [10].

## 5. Conclusions

Under the operating conditions of this study (continuous stirring by circular motion at 20 rpm at room temperature), we can conclude from the regression equations that K2EDTA is stable throughout the 3 h study period, citrate is stable for 19 min in impedance and 22 min in fluorescence, and MgSO_4_ is stable for 30 min in impedance and 24 min in fluorescence.

The limitations of this study are that it does not reflect the wide variation in tube transport conditions. The conclusions of this study will have to be specified by varying stirring conditions, stirring time, and temperature. In any case, given the platelet aggregation favored by agitation of citrate and MgSO_4_ tubes, we can already recommend that when citrate or MgSO_4_ tubes are used, they should be transported in a vertical position, avoiding any agitation, so as not to give falsely low platelet results, whatever analyzer is used.

## Figures and Tables

**Table 1:** Hodge-Lehman pseudo-median of platelet difference percentage (PMed) from 70 patients sampled on K2EDTA, citrate, or MgSO_4_ analyzed on XN10 compared with T0 as a function of the number of revolutions. Stirring is performed on a 20 cm diameter disk rotating at 20 rpm. *p*-values are calculated either with a two-side test (Wilcoxon) to test equivalence with respect to the desirable EFLM bias (4.5%) if PMed is less than the bias (shown in bold) or with a 2-side sign test (Wilcoxon) if PMed is greater than the bias. **Abbreviations:** CI: PMed 95% confidence interval; Imp.: impedance; Fulo.: fluorescence; revs: revolutions.

PMed (%)	300 revs (15 min)	600 revs (30 min)	1200 revs (60 min)	1800 revs (90 min)	2400 revs (120 min)	3000 revs (150 min)	3600 revs (180 min)
EDTA Fluo.	**0.38**	**0.51**	**0.63**	**0.67**	**1.11**	**1.13**	**1.17**
(CI)	(0.08, 0.69)	(0.24, 0.80)	(0.30, 1.00)	(0.31, 1.05)	(0.66, 1.71)	(0.68, 1.63)	(0.68, 1.68)
*p*-value	<0.0001	<0.0001	<0.0001	<0.0001	<0.0001	<0.0001	<0.0001
Citrate Fluo.	−10.55	−13.92	−16.32	−19.76	−24.11	−26.79	−29.76
(CI)	(−12.44, −8.29)	(0–16.86, −11.66)	(−19.62, −13.59)	(−23.48, −16.51)	(−28.19, −20.72)	(−31.17, −22.99)	(−34.39, −25.31)
*p*-value	<0.0001	<0.0001	<0.0001	<0.0001	<0.0001	<0.0001	<0.0001
MgSO_4_ Fluo.	**−2.66**	**−4.47**	−9.69	−15.79	−22.29	−27.84	−33.18
(CI)	(−3.49, −1.98)	(5.91, −3.35)	(−11.83, −7.33)	(−19.38, −12.24)	(−26.61, −18.14)	(−32.20, −23.60)	(−37.72, −28.92)
*p*-value	<0.0001	0.48	<0.0001	<0.0001	<0.0001	<0.0001	<0.0001
EDTA Imp.	**0.65**	**0.96**	**1.43**	**1.17**	**1.50**	**2.18**	**2.26**
(CI)	(0.11, 1.39)	(0.25, 1.65)	(0.72, 2.11)	(0.50, 1.93)	(0.77, 2.25)	(1.44, 2.95)	(1.50, 3.07)
*p*-value	<0.0001	<0.0001	<0.0001	<0.0001	<0.0001	<0.0001	<0.0001
Citrate Imp.	−12.36	−18.54	−20.64	−24.59	−29.36	−31.51	−33.92
(CI)	(−16.96, −7.37)	(−23.20, −14.04)	(−25.38, −16.17)	(−29.43, −20.34)	(−34.38, −24.58)	(−36.66, −26.75)	(−36.68, −28.81)
*p*-value	0.0012	<0.0001	<0.0001	<0.0001	<0.0001	<0.0001	<0.0001
MgSO_4_ Imp.	−2.42	−3.81	−6.95	−11.41	−16.33	−21.25	−25.49
(CI)	(−3.14, −1.69)	(−4.99, −2.83)	(−8.74, −5.54)	(−14.12, −9.31)	(−19.92, −12.99)	(−24.93, −17.59)	(−29.51, −21.93)
*p*-value	<0.0001	0.1134	0.0007	<0.0001	<0.0001	<0.0001	<0.0001

## Data Availability

The data that support the findings of this study are available from the corresponding author upon reasonable request.

## Supplementary Materials

The following supporting information can be downloaded at: https://www.mdpi.com/article/10.3390/jcm13185350/s1, Table S1: Platelet between run imprecision; Table S2: mean relative bias of sodium citrate and MgSO_4_ compared with K2EDTA
