# Supplementary material for: Impact of Shaking EDTA, Citrate, or MgSO4 Tubes on Platelet Count Results
Source: J Clin Med. 2024 Sep 10;13(18):5350. doi: 10.3390/jcm13185350 (PMC11432049; doi:10.3390/jcm13185350)
Supplement: Supplementary file 1 [file jcm-13-05350-s001.zip › Table S1.pdf]

Table S1

Platelet between run imprecision during the study with the quality control materiel obtained by Sysmex

**Parameters**	**Low Level**<br>**Mean**<br>**%CV**	**Normal Level**<br>**Mean**<br>**%CV**	**High Level**<br>**Mean**<br>**%CV**
**PLT-I 10^9/L**	82<br>3.1	246.3<br>2.1	559<br>1.5
**PLT-F 10^9/L**	79.7<br>2.9	245<br>2.1	542.4<br>1.4
