# Supplementary material for: Impact of Shaking EDTA, Citrate, or MgSO4 Tubes on Platelet Count Results
Source: J Clin Med. 2024 Sep 10;13(18):5350. doi: 10.3390/jcm13185350 (PMC11432049; doi:10.3390/jcm13185350)
Supplement: Supplementary file 1 [file jcm-13-05350-s001.zip › Table S2.pdf]

Table S2

Mean relative bias in percentage and 95% confidence interval (in brackets) of citrate or MgSO4 platelets from 70 patients at T0 compared with K2EDTA.

The bias below the desired bias of the EFLM is shown in bold.

Mode	Citrate	MgSO4
Impedance	-20%<br>(-17.6% ; -22.4%)	-11.8%<br>(-9.7% ; -13.9%)
Fluorescence	-10.5%<br>(-9.6% ; -11.3%)	**-2.9%**<br>**(-2.2% ; -3.7%)**
